# Ten simple rules for principled simulation modelling

**DOI:** 10.1371/journal.pcbi.1009917

**Published:** 2022-03-31

**Authors:** Laurel Fogarty, Madeleine Ammar, Thomas Holding, Adam Powell, Anne Kandler

**Affiliations:** 1 Theory in Cultural Evolution Lab, Department of Human Behavior, Ecology, and Culture, Max Planck Institute for Evolutionary Anthropology, Leipzig, Germany; 2 BirthRites Research Group, Department of Human Behavior, Ecology, and Culture, Max Planck Institute for Evolutionary Anthropology, Leipzig, Germany; 3 Waves Research Group, Department of Human Behavior, Ecology, and Culture, Max Planck Institute for Evolutionary Anthropology, Leipzig, Germany; Carnegie Mellon University, UNITED STATES

This is a *PLOS Computational Biology* Methods paper.

## Introduction

Learning to model can be simultaneously exciting and frustrating, and people’s “first models” often suffer from very similar conceptual and technical problems. Sometimes, these are so severe that the model seems impossible to analyse, can’t generate interpretable results, or simply can’t be published. Here, we don’t aim to teach the technicalities of modelling—each field will have its own technical requirements, idiosyncrasies, and conventions, and most of this is well covered elsewhere (for example, see [1] for an introduction to modelling in evolutionary biology). Instead, we focus on softer skills involved in modelling—some guiding principles of model design, implementation, and reporting that we hope provide a concise and constructive guide to identifying and avoiding the most common pitfalls and producing robust, useful, and interpretable scientific models in general. In what follows, we deal with discrete event simulation and agent-based models (see, e.g., [2,3]). These are frequently used scientific models applicable to systems where stochasticity plays an important role. Simulation models can seem more accessible than formal analytical models where the need for mathematical training is more obvious. Yet, despite their accessibility, building, analysing, and deriving meaningful inferences from these models is not straightforward and requires researchers to develop and maintain a broad skill set—from philosophical understanding and mathematical clarity to clear figure design.

The following 10 rules constitute an informal and general best practice guide to scientific simulation modelling and appear roughly in the order in which issues might pop up in the course of a modelling project. They focus on conceptual issues (Rules 1 to 3), general technical and coding issues (Rules 4 to 7), and analysis and presentation of modelling results (Rules 8 to 10).

### Rule 1: Ask a specific question, and know your aim

Ideally, the start of a modelling project would be a clear idea of what, specifically, you want to understand and a clear understanding of how a model’s output can be used to this end. In fact, the term “model” is often defined in terms of its aim. For example, Minsky [4] defines a model in terms of its usefulness in answering questions about a target system: “To an observer B, an object A* is a model of an object A to the extent that B can use A* to answer questions that interest [them] about A”. Often, though, crystallising this aim is surprisingly difficult, and many new modellers start out with a sense that a model would be useful and that more theory is generally helpful, but without a clear recognition of how or why. This feeling is the first step in a multistep process that precedes a model, but actually beginning to write a model at this stage is likely to produce a directionless and vague description of a system that is difficult to mine for insight post hoc.

Of course, it is not necessary to have a full understanding of the epistemology of model building before starting, but some philosophical ideas are relevant to choosing a modelling approach that matches a project’s aims. Different types of models have different explanatory powers (e.g., [5,6]), and it is important to identify what kind of insight you are aiming for and what kind of models are capable (or incapable) of providing it. For example, phenomenological models capture something about the relationship between, say, an input and an output. While they can recreate important patterns in data and can often be used for prediction (by which we mean precisely forecasting new or future observations [6]), they need not provide any mechanistic insight and have limited explanatory power. A good and often cited example of this kind of model is Ptolemy’s model of planetary motion [7], which described the movements of the planets, and could be used to predict the behaviour of the solar system. So, using this model, you might look north in the sky tonight at 11 PM, and know precisely what you should see. But the model provides no explanation as to why the solar system behaves in this way. In contrast, Einstein’s theory of general relativity, for example, offers a more general explanation of why large bodies like the sun and the planets are attracted to one another and how and why they orbit. This theory provides a fuller mechanistic explanation of the solar system than Ptolemy’s and concerns itself with both prediction and these general insights. Models of evolutionary processes are similar—they often focus on mechanistic insight, as scientific researchers are often most interested in “why” questions, and many branches of evolutionary theory were also developed with real-world, precise prediction in mind (see, e.g., [8]; see also [9] and citations therein for a discussion of prediction in evolution). So, which kind of model you make will depend, then, on the level of explanation needed and the use to which the model will be put.

### Rule 2: Build a model; don’t try to replicate the entire system

Researchers diving into modelling from a platform of substantial, detailed knowledge of their system often baulk at the idea that certain, even seemingly important, elements of the system can be unhelpful to model and so can be productively excluded. George Box [10] famously said that all models are wrong, but some are useful. What he meant was that all models are abstractions from reality, and, contrary to intuition, a closer approximation to reality does not always make a better model. A useful illustration here is a map [11,12]—just as a model is designed to generate some carefully considered insight into a system, maps are designed to answer the question “how do I get from A to B?” Although more detail on a map makes it more accurate, technically speaking, it might also be a considerable hindrance. Instead, the most useful maps are severe abstractions from reality that only include the bare minimum detail needed to answer the question—road names, directions, and landmarks. An excellent visual example of this can be seen in the famous map of the London Underground system produced by the Transport for London (TFL; the London transport authority) and initially designed by the draughtsman Henry Beck in 1933 [13]. The map produced before 1933 (Fig 1 in [13]) was technically accurate and certainly more geographically detailed than its more abstracted counterpart (Fig 2 in [13]). However, it is an updated version of this second map that is given to millions of tourists travelling across London every year. The inclusion of only important information, abstraction, and omission of extraneous geographical detail has made it one of the most famously useful graphics in history. Models of complex systems should be the same—as detailed as needed but no more detailed than that.

So, if you shouldn’t model the system you know in complete detail, what should you model instead? This depends to a great extent on the type of model that you have decided to make (Rule 1). Assuming that some mechanistic insight is the aim, based on knowledge of the system and precise formulation of the research question, a good strategy is to identify putative interactions or mechanisms that could underlie the behaviour or phenomenon under investigation and model those mechanisms. In other words, it is sensible to propose an engine that could be driving the phenomenon of interest and then make characterising and investigating that engine the central focus of the model. Anything in the system irrelevant to that engine is irrelevant to the model. Part of the art of modelling is identifying putative mechanisms and ensuring that the logical thread between mechanism and behaviour is unbroken.

### Rule 3: Be rigorous, and allow the time to be rigorous

Perhaps this is an obvious rule—cience and rigour usually go hand in hand. Successful modelling requires considerable attention to mathematical and computational detail. It is vital that all concepts and all code used in a simulation model are clear to the author and rigorously tested—from their construction to their downstream implications (see Rule 6). Using convenient mathematical or statistical concepts with little or no knowledge about their inner workings will likely lead to their misuse and maybe to serious fundamental errors. Similarly, it can be dangerous to use, for example, software packages or useful-looking chunks of code as untested black boxes (but see Rule 6). The assumptions of the methods implemented in the code, and the implementation itself, must be clear and consistent with the assumptions and aims of your model. Alongside the mathematical and technical details of each part of the model, careful consideration should be given to the order in which these parts or processes are executed and what effect that ordering (or scheduling) has on the output.

All of this is to say that modelling decisions need careful thought and, as with empirical data analysis, designing a model that describes your system in adequate (but not excessive) detail, and deriving robust inferences from it, can take significant time. This is time which needs to be budgeted for in any simulation modelling project from the outset.

### Rule 4: Have a plan for analysis

The aim of this rule is to help you to avoid a surprisingly common and deeply frustrating situation: creating a monster simulation with so many moving parts that it just can’t be analysed meaningfully—a situation where you could say to yourself something like “I’ve finished coding this simulation, but how on earth can I analyse so many interactions and so many outputs?” Reaching this point probably means that something has gone wrong in the implementation of Rules 1 or 2—or that the model has been coded without a good plan for its analysis. So, perhaps you know the question you want to answer, you know or have guessed the relevant aspects of the system, but you didn’t plan how the model can answer the question. In general, it is important to have an idea of the analysis steps necessary to answer aspects of the central question. These plans are flexible and develop throughout a project, of course, but an initial plan is crucial.

Your exact plan for analysis will depend on your aim and the kind of model you have constructed. For example, if you are interested in unravelling mechanistic relationships, you might use simulated data to explore the workings of the model and to analyse exactly how different parameters or processes affect the outcome. To do this successfully, the number of parameters and processes should be manageable (see Rule 2), and parameter explorations should be principled (see Rule 8).

Or, if you are interested in fitting your model to observed data, you may need to consider exactly how the model output maps to existing and future data and identify appropriate statistical techniques to robustly test between models and estimate parameters. Here, it will be important to be aware of the limitations and assumptions of the statistical techniques in addition to those of the model itself (see Rule 3).

### Rule 5: Be kind to your future self (and your readers), and minimise opportunities for errors

You will need to revisit your code, sometimes many months after the analysis has been done and the paper has been written. This can either be a difficult exercise for your future self or a simple matter of glancing at your structured, documented, and organised code. Of course, it is best to aim for the latter. This means that code needs to be well structured and well commented so that you are able to easily reconstruct what has been done. To this end, this rule advises a number of good coding practices that are particularly important for simulation models, which can become large and unwieldy projects very quickly. All of these practices will benefit the readers of your model as much as they benefit your future self.

First, the names of variables, functions, and parameters should be intuitive, consistent, and immediately intelligible. This makes the code more readable and minimises unnecessary comments. Similarly, adopting consistent formatting conventions (such as indentation and other whitespace), much like punctuation in a sentence, improves code readability. Commenting should be done with both your future self and your future readers in mind. For larger-scale multiresearcher and multiyear projects, more detailed documentation should be created and developed in tandem with the code.

As a project grows, it becomes difficult to keep track of the interactions and dependencies between the different parts of the code. This complexity can be made more manageable by giving your code a well-defined and modular structure, meaning that sections of code should be divided into logically related units and the interfaces between these units of code should be well defined. Different programming languages offer different tools to achieve this, and the following advice applies equally to all of them. We’ll use the concept of functions as an example because this is the most fundamental and most commonly used. The aim here is to identify units of self-contained logic and create functions that encapsulate this logic. Functions should have a single clear purpose so, if you find you’re writing a function which performs multiple conceptual actions, consider breaking it up into 2 or more functions. Encapsulating logic in this way creates higher-order building blocks that can be reused and avoid code duplication. This reduces the opportunity for errors, not only because there will be less code to begin with, but also because each logical unit can be easily tested individually.

In fact, it is good practice to test functions as you write them—this helps to avoid bugs in “low-level” functions that often manifest as difficult to diagnose problems downstream. The ubiquitously useful coding mantra “fail early and fail loudly” posits that it is better to raise an error as soon as it could feasibly be detected. So, for example, it is best to have functions check that their inputs are valid rather than allowing them to blindly compute and pass nonsensical output downstream.

Despite taking these steps to manage the complexity of your project, you will write bugs. Everyone does and it is unavoidable. But, if you have written modular, well-structured code, it will be much easier to test the code, find the bugs, and squash them.

With a similar aim in mind, it is now common practice and explicitly required by some journals that model code is published alongside the paper it generates. This has a number of important benefits. It facilitates open and reproducible science. It also benefits you as a modeller. Bugs and coding issues can be identified by reviewers and readers who go through the code. This can be an important source of error checking.

Finally, but equally importantly, you will need to test that the (now hopefully bug free) code actually does what it is supposed to in terms of the model’s dynamics. The full code, and each constituent part, should be put through its paces using simple test cases for which you have either good intuitions or solid analytical expectations to which the model outcomes should conform.

### Rule 6: Write practical code

Practical code doesn’t have to be perfect, fully optimised, or even beautifully written, but it is fit for purpose, well tested, and clearly documented so that others (and you) can have confidence in it (see Rule 5). Practical coding also means using your coding time efficiently. Investing some time in learning to use the debugging tools available to your programming language will quickly pay for itself in time saved identifying and fixing bugs more quickly. There are a great many well-tested and already optimised scientific libraries/packages, and there is usually nothing to be gained by reimplementing functionality that already exists. Making use of good quality libraries will speed up development and reduce your opportunities to write bugs (see Rule 5), although it is critical to ensure that the methods used are appropriate for your context (see the note about the dangers of “black boxes” in Rule 3).

Fit-for-purpose code runs in a reasonable amount of time. Many parameter sweeps with many repetitions of stochastic simulations will be necessary to generate robust, publishable results (see Rule 7), so it’s very likely that you’ll need to perform some optimisation to make your model run more quickly. There is some discussion about the best time and way to make code efficient. Conventional wisdom often attributed to the programming pioneer Donald Knuth states that early optimisation is problematic when a lot of time might be wasted making the wrong parts of the code faster. This issue of timing does not change the fact that, for simulation models, writing efficient code is important, but it does mean that making the right parts of the code efficient is key. A common approach is to write relatively quick “prototype” code that emphasises correctness, good structure, and readability and then identify potential bottlenecks by using profiling tools to see how much time is spent running each part of your code. When it comes to actually implementing optimisations, understanding the intricacies—and potentially underexploited capabilities—of your particular programming language helps enormously. For example, large speed increases can be gained in R, Python, and MATLAB by vectorising code that relies heavily on loops, but this may not be the case, for instance, in C++ under the same circumstances. In general, developing a good understanding of the strengths and weaknesses of the data structures provided by your programming language allows you to make informed decisions about how to best temporarily store, permanently save, and manipulate information.

### Rule 7: Don’t ruin your efforts; run the simulation in a methodical way

Once your model is constructed, it may be tempting to think that you are on the home stretch. As with model construction, though, running a simulation is fraught with difficult decisions that require robust justifications. How exactly will the model be run? How will it be initialised? How long should it run for? How many iterations are enough? Unfortunately, usually, there are no hard and fast rules to help with any of those questions; the answers depend on the model, and it is up to the modeller to decide and to demonstrate that the decisions lead to robust results.

Initialising a model is not always straightforward. For many models, it is best to randomly set initial states or parameter values—perhaps drawn from a prior distribution. Alternatively, it may be possible to start from some deterministic point, potentially informed by empirical observations. Either way, you need to be sure that the outcome of the model is not obscured by the initial conditions. This might require, for example, a sufficiently long burn-in time, such that the model reaches a state where meaningful comparisons between outcomes can be made—this could be at an equilibrium point or some other predefined state that is appropriate for the specific model (e.g., when a critical minimum threshold is reached). Additionally, a full characterisation of the system may involve a thorough sweep of initial conditions.

Simulation models often have many moving parts (but hopefully not too many; see Rule 2) and probably a number of stochastic elements. So, in order to comprehensively explore the space of possible outcomes, then, the model needs to be run a large number of times to fully capture the effects of the model parameters as well as stochasticity (another good reason for efficient model code; see Rule 6). What “large” means here depends on the model and only the modeller can decide and demonstrate that the space of possible outcomes has been meaningfully explored. When there is a stochastic element to the simulation, single runs are largely uninformative—understanding and quantifying the means and distribution of outcomes is what’s important (Rules 9 and 10).

### Rule 8: Find and report the model’s limits

One of the most underappreciated parts of analysing and writing up a simulation model is breaking it and carefully reporting the many ways in which you can break it. Readers need to know when the dynamics or data the model produces are outside what’s realistic for the system and thus when the model can and cannot contribute to their understanding of the system. In other words, the model may produce nice results for some parameter combinations but reporting this is not enough; modelling papers need to report the circumstances under which the model works and the circumstances under which it stops making sense—where it breaks down. Typically, this involves an exploration of the parameter space. This is achieved by running the model over a reasonable spread of parameter values. What is reasonable here is often constrained by empirical estimates, physical reality, or mathematical concepts. For example, proportions need to be in the range [0,^1^], temperatures compatible with animal life are within a specific range, etc. This comprehensive but constrained parameter sweep is a principled way to show the parts of parameter space for which the model generates realistic dynamics, and, equally importantly, the parts of parameter space for which it does not—and why.

It might be tempting to see reporting this kind of model failure as decreasing the value of the work, but the opposite is true. Reporting detailed information on where the model breaks down hugely increases its value and the confidence that readers can have that the inferences you draw are not just the result of cherry picking parameter values or arbitrarily reducing the parameter space post hoc.

### Rule 9: Make honest interpretations

This follows from and builds on the previous rule. Rule 8 says that substantial effort should be dedicated to finding the limits of a simulation model’s technical utility. This rule suggests that this search for the model’s limits extends to its conceptual robustness, too. For example, if the model can only give one result—even if it is correct as far as the aims of the project are concerned—the model is not meaningful and is likely to be uninformative when confronted with data. There are limits to the usefulness of any model and understanding what you can accurately and confidently infer from the model and what is out of reach is the basis of good interpretation. It is the author’s responsibility to ensure that this information is clearly communicated to the reader and that the limits of inference, given the assumptions, are carefully explained. This is, at the end of the day, a matter of honesty in interpretation and honesty in reporting.

Models live or die on their explicit and implicit assumptions and these need to be made unambiguously clear. Listing explicit assumptions, although sometimes overlooked and often difficult, is easier than the more daunting task of examining all implicit assumptions built into the model and their impact on the dynamic. Once this is done, any model interpretation must be tightly bound to those assumptions. The literature is full of examples of slippage where assumptions were carefully explained in a methods section but, later, no longer constrain interpretation. This can easily lead to overinterpretation, i.e., results are interpreted outside the range of explored and reported parameters, and conclusions are drawn that are not justified.

It is also good to be clear, with yourself and your readers, that producing a plausible model is not the same as producing a correct model. This distinction is discussed in philosophy of science as the difference between “how possibly” and “how actually” models (see, e.g., [14,15]). In other words, when it comes to the comparison of relevant model outputs with data, finding one model that generates data consistent with what is observed does not mean that it is the unique causal model. This is also known as the problem of equifinality (e.g., [16]). Often, a range of models can be identified as responsible for the same patterning in observable data. To be able to claim that you have the unique causal model, you need to show, at the very least, that all other a priori equally plausible models do not fit the data equally well. This might not be possible, and you might have to accept that, given available data, there are a number of alternative plausible models or explanations. This is fine and expected. Often, in complex open systems, it is clearly exaggerated claims of the unique supremacy of an author’s model that sounds alarm bells.

### Rule 10: Clearly communicate model results—informative figures are key

Clearly summarising model outputs or simulated data involves showing outcomes across the chosen parameter space—where the range or limits of this is robustly justified (see Rule 8)—and for multiple, rather than single runs (see Rule 7). To summarise multiple runs of a simulation, it is often advisable to go beyond just mean values—understanding the range of possible outcomes is itself informative. It is often highly instructive to use a range of metrics to describe these outcomes or even show the raw distributions themselves which can offer insights and suggest interpretations that are obscured by summary statistics. It is only in this way that you can discover anomalies like bimodal results or a large spread of outcomes that defy interpretation.

Communication of models and modelling results can be a difficult balance. If the work is interdisciplinary in the sense that it will be read by both fellow modellers and nonmodellers, it is important to bear in mind the level of technical expertise of all readers. You should aim to provide details in a form that is understandable and digestible to those who, for example, might want to reproduce and extend the model itself and to those who wish to make use of its insights but are not modellers themselves. In other words, some effort should be made to enable readers of all backgrounds to follow (and critique) the logic and implementation of the model as far as possible.

Clear and informative figures play a huge role here in communicating both the results from, and limits to, a model. Figures are often the way that readers will first encounter the model and its results, and the more useful, readable information they contain, the more your readers can assess about the utility of the model at a glance.

## Conclusions

We will conclude by saying 2 things. First, even with some experience, all of the authors of this manuscript have broken some or all of these rules at some point. We have all made less-than-ideal figures, written models with too many parameters, or started a model with only vague plans for how it will be analysed. Every novice modeller will make these mistakes—it is part of the process of learning to model. Sometimes, muddling through these difficulties is easier with a guide and that leads us to this last piece of advice: Don’t be afraid to talk to a modeller with training and experience early in a modelling project. Problems with any of the conceptual or technical points mentioned above should be quickly identified by someone who makes models every day. They will have an intuition for the feasibility of the project’s aims, analysis, and the suitability of the model—this is one of those nebulous, hard-won skills that comes from years of accidentally breaking the rules of model building.

Finally, the technical capability to make and analyse useful and informative models now sits on almost every researcher’s desk. As more researchers from diverse backgrounds embark on modelling adventures, many scientific fields will experience a welcome and necessary push towards theory that is more closely linked to empirical research, which will ultimately contribute to deeper scientific insights. Our aim in writing these 10 rules is to help more new modellers to start with a clearer understanding of the modelling process and, hopefully, with more confidence in the outcome of their theoretical projects.
